# Detailed HIV Self-Testing Patterns Derived from Paradata in the mLab App Clinical Trial

**DOI:** 10.1007/s10461-025-05013-1

**Published:** 2026-01-09

**Authors:** Thomas F. Scherr, Austin Hardcastle, Carson P. Moore, Dheemanth Majji, Lisa M. Kuhns, Robert Garofalo, Rebecca Schnall

**Affiliations:** 1 Department of Chemistry, Vanderbilt University, Nashville, TN USA; 2 Division of Adolescent and Young Adult Medicine, Ann & Robert H. Lurie Children’s Hospital of Chicago & Department of Pediatrics, Feinberg School of Medicine, Northwestern University, Chicago, IL USA; 3 Columbia University School of Nursing, New York, NY USA

**Keywords:** Mobile health, HIV self-testing, Paradata

## Abstract

Self-testing is a critical component of public health initiatives aimed at slowing and stopping the spread of HIV. It has the promise of accessibility, reliability, and convenience, and because of the benefits derived from its inherent privacy, self-testing may overcome the barriers associated with HIV screening with at-risk populations. Still, questions remain about whether and how self-testing can adequately link patients to care and engage them with other interventions when needed. This presents an opportunity for digital platforms to bridge the gap, connecting patients with the HIV continuum of care. During a recent clinical trial of the mLab App, a mobile health intervention designed to increase HIV testing rates, we collected screen-level paradata—detailed logs of user interactions within the application—focusing specifically on user behavior during the test-result interpretation workflow. Among enrolled participants, 330 HIV self-tests were completed in the app, with 74.2% occurring within the scheduled testing window. Three post-timer screens (Preview Test, Upload Picture, and Visual Result) accounted for 60.6% of incomplete testing sessions, highlighting friction points in the result interpretation workflow. Users who experienced discordant automated results (i.e., when the app’s automated interpretation differed from the user’s visual inspection) demonstrated reduced subsequent engagement but did not significantly alter future test-taking behavior. These findings identify critical moments in the HIV self-testing workflow and provide actionable insights for improving the design of digital tools that support accurate testing and linkage to care.

## Introduction

HIV remains a critical public health issue globally, with nearly 40 million people living with HIV [1]. An estimated 5.4 million people globally were unaware that they were HIV-positive [1]. Given the successful scale-up of services available for those living with HIV over the last two decades [2], it is clear that HIV screening is a necessary gateway to diagnosis and treatment. Still, screening globally, as in the United States, faces challenges of reaching those groups most at risk of HIV, including men who have sex with men (MSM), transgender women, intravenous drug users, and sex workers [3]. In the US, HIV incidence rates are further elevated among racial and ethnic minorities [4]. These communities are a difficult-to-reach at-risk population, due to complex and intertwined public health and socioeconomic factors (i.e., stigma, food insecurity, housing availability, substance misuse) [2]. In this context, HIV self-testing (HIVST) emerges as a solution [5]. By offering privacy, convenience, and accessibility, HIVST has the potential to increase testing rates among difficult-to-reach populations [6–9]. 

Despite its benefits, HIVST poses potential challenges that impede its wider acceptance. One primary concern is that self-testing might diminish interactions with healthcare providers, where more sensitive diagnostic tests are available and additional preventive measures could be administered [5]. Another significant issue is that individuals who obtain a reactive or preliminary positive result from a self-test may be less inclined to pursue confirmatory testing and subsequent linkage to appropriate care [8, 10]. Moreover, the psychological impact of receiving a reactive test result without immediate professional support is a notable challenge [9]. Stakeholders have also raised concerns about the potential for misinterpreted results and the accuracy of self-testing, especially due to the lack of training or experience in point-of-care diagnostic evaluation [11]; however, these issues may be less impactful in practice than other barriers [12].

**Fig. 1:**
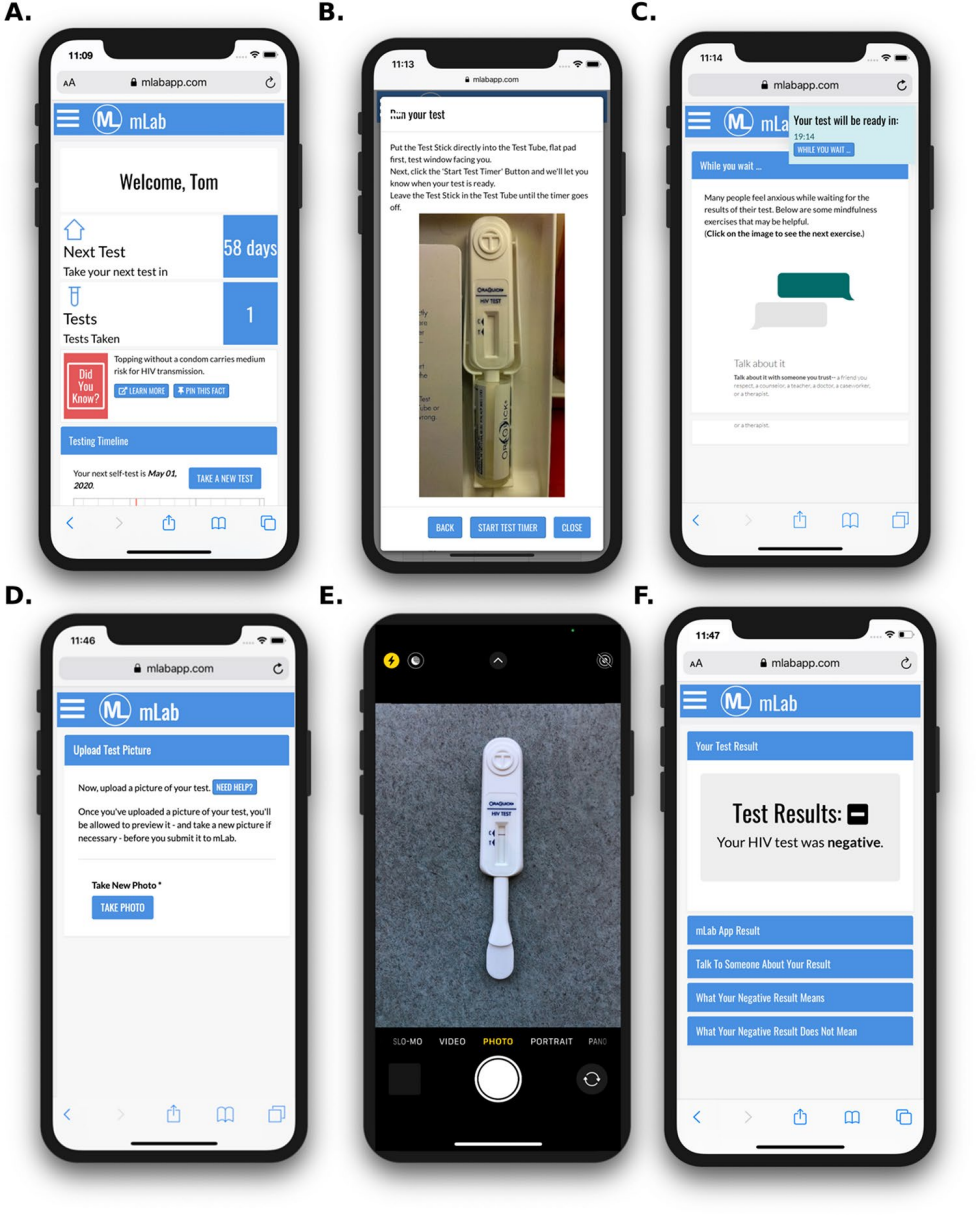
Screens from the mLab App showing the HIV self-testing workflow. **A** Landing/home screen displaying user status, next testing window, and testing timeline. **B** Guided walkthrough with test instructions prior to initiating the timer. **C** Countdown timer overlaid on mindfulness exercises during the waiting period. **D** Prompt for users to upload a photo of their completed test. **E** Native device camera interface used to capture the test image. **F** Test results screen displaying the interpreted result (negative example shown) with explanatory information about what the result means and does not mean

Digital health tools represent a potentially transformative approach to overcoming these barriers [8, 13–18]. One such platform, the mLab App, was developed specifically to support HIVST (Fig. 1). It combines digital technology with HIV home tests and guides users through the testing process with step-by-step instructions and provides clear interpretation of results. Moreover, it ensures that individuals with positive results are efficiently linked to care services, thereby bridging critical gaps in the self-testing pathway. Digital health intervention design frameworks emphasize usability, engagement, and feedback as central to achieving behavioral and clinical outcomes [19–21]. The mLab App was designed with these principles in mind, integrating behavioral scaffolding and digital feedback mechanisms to support each step of the self-testing process. Furthermore, the collection and analysis of paradata offers a tool to better understand and optimize the design of these behavioral and digital health interventions [22, 23]. Integrating paradata into HIV self-testing applications such as mLab can reveal points of user difficulty and support iterative improvements that enhance engagement and accuracy.

The efficacy of the mLab App was evaluated through a multi-site randomized clinical trial conducted over three years in New York City and Chicago. While the parent trial assessed overall HIV self-testing uptake and PrEP use among 522 (211 in the mobile app arm) young men who have sex with men (YMSM), the present analysis focuses specifically on screen-level paradata captured during HIV self-test result interpretation within the app. This secondary analysis examined user behavior during the interpretation workflow, including the timing of test initiation, the frequency of incomplete sessions, and actions following discordant automated versus visual results. Detailed results of the parent randomized controlled trial, including HIV self-testing uptake, linkage to care, and PrEP outcomes, are reported elsewhere [24]. However, prior studies have not characterized the moment-to-moment behaviors that occur within the self-testing process itself—particularly where users disengage or deviate from intended workflows—leaving a critical gap in understanding how digital tools can better support accurate test completion and linkage to care. The current manuscript builds upon that work by analyzing the paradata component of the mLab arm to characterize user behavior during test-result interpretation.

While our other work focuses on the general analysis of paradata collected during the study [25], which reveals detailed patterns of user interactions with the mLab App, this manuscript focuses specifically on paradata related to usage of the interpretation of test results functionality in the mLab App. Through this detailed examination of the dataset, this manuscript will contribute valuable insights into the optimization of digital health tools for HIV testing. This work can provide a deeper understanding of how user behavior impacts the efficacy of self-testing applications, and how these insights can be leveraged to improve health outcomes in digital health practices.

## Methods

### Study Design, Participants, and Data Collection

**Fig. 2:**
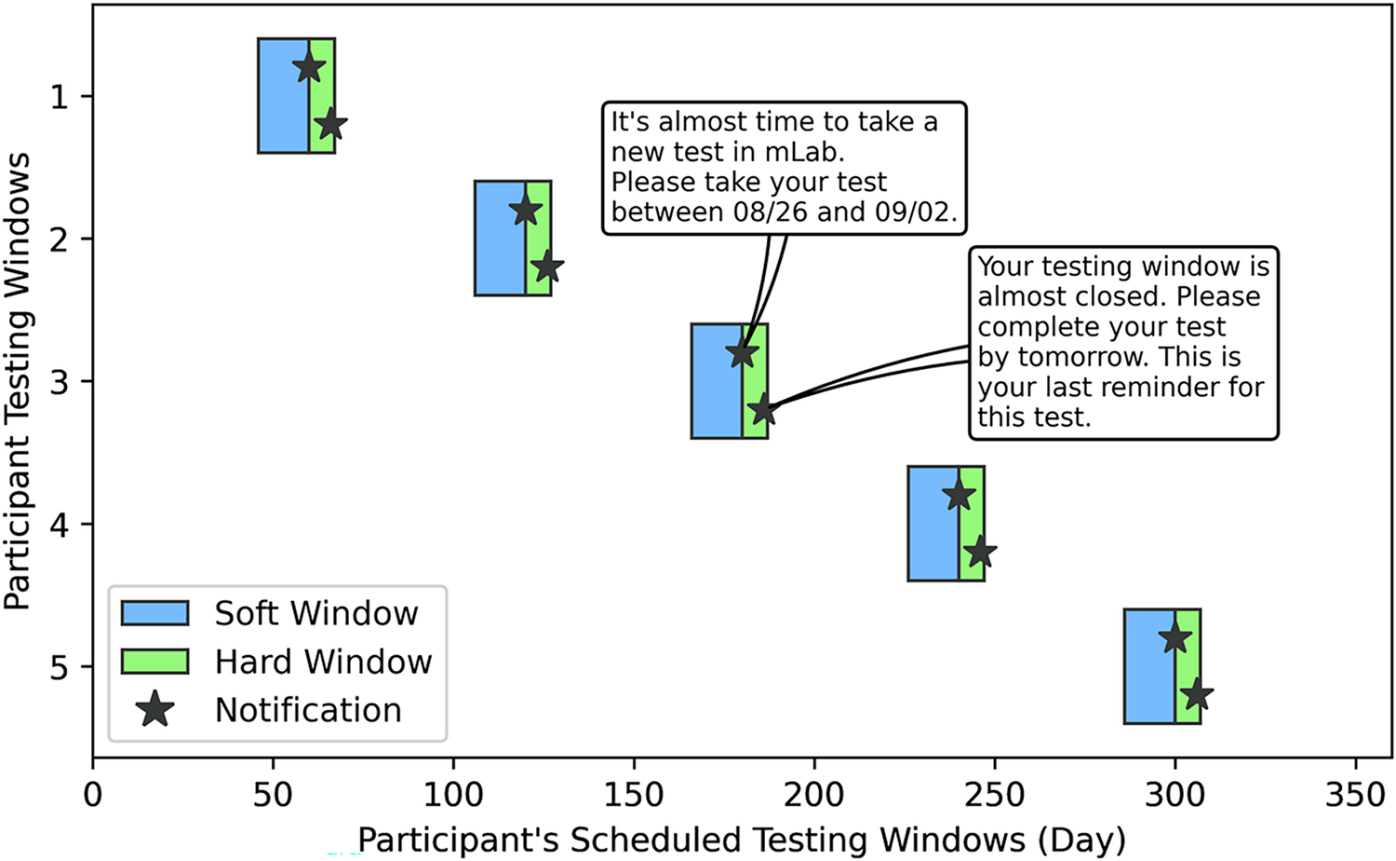
Participant’s scheduled testing windows and timing of notifications

The study design, participants, and data collection methods have been described in detail in our other work [24–26]. Briefly: The mLab App was evaluated as part of a multi-site randomized clinical trial conducted over three years in New York City and Chicago to assess HIV self-testing uptake and PrEP usage among 522 YMSM participants (*n* = 211 in the mLab App arm). This manuscript reports a secondary analysis of the paradata component of that trial, focusing on user interaction patterns during the HIV self-test interpretation sequence rather than trial-level behavioral outcomes. At the time of their enrollment, each mLab App arm participant was assigned 5 one-week-long testing windows, referred to as hard windows. They were sent a text/email message, depending on their preference settings, two weeks prior to the start of their hard window; the time from two weeks prior to the start of the hard window was referred to as the soft window (Fig. 2). Paradata were captured through event-level logging of user interactions (e.g., screen views, button clicks, form submissions, and photo uploads) using a custom asynchronous JavaScript library interfacing with a custom PHP API. This approach ensured data were recorded in real time without interrupting the user experience. All paradata were de-identified with coded user ids and transmitted via encrypted channels to a secure study REDCap [27] project, ensuring that no personal identifiers or image data were stored within the paradata stream. The technical implementation is described in detail elsewhere [25]. Multiple rounds of usability testing were conducted to refine the app’s interface and functionalities based on feedback from the target demographic, enhancing usability, engagement, and data accuracy.

Participants in the mLab arm are described in detail elsewhere [24, 28]. Briefly, young men who have sex with men (YMSM) aged 18–29 years (mean = 24.3, SD = 3.2), recruited from community organizations and online advertisements in New York City and Chicago. The sample was racially and ethnically diverse, including 58.3% White, 11.8% Asian, 10.4% Black/African American, 10.0% multiracial, 5.7% identifying as ‘something else,’ and 0.9% American Indian/Alaskan Native. Roughly one-third (34.6%) identified as Hispanic or Latino. Participants represented a broad range of educational attainment, with 7.6% not finishing high school, 15.2% completing high school or GED, 23.7% having some college or technical training, 26.1% holding a bachelor’s degree, and 25.6% holding a graduate degree.

### Data Visualization and Statistical Analysis

Custom Python scripts utilizing Pandas, Matplotlib, Seaborn, Scikit-Learn, and SciPy were developed to analyze and visualize the testing paradata in the mLab App. The raw paradata, a clean de-identified version of which is deposited at 10.6084/m9.figshare.28211636, includes user IDs, timestamps, accessed pages, and screen interactions within the app (i.e., button presses, menu opening/closing). Descriptive statistics were performed on filtered and derivative datasets to generate summary statistics for the variables, including count, mean, standard deviation, quartiles, and extrema. Pearson correlation coefficients were calculated to examine the relationships between continuous variables such as age, session count, test count, total time, health literacy, health engagement, and usability [29] at the last survey time point. To evaluate the normality of distributions within the dataset, the Shapiro-Wilk test was employed, where a p-value less than 0.05 indicates a departure from normality. Levene’s test was used to assess the homogeneity of variances across the distributions of different groups, with a p-value less than 0.05 indicating significantly different variances. For comparing distributions across different independent groups, the Kruskal–Wallis test was utilized to compare group medians. This non-parametric alternative to ANOVA was chosen because the Shapiro–Wilk and Levene’s tests indicated that several variables violated assumptions of normality and homogeneity of variance. The Kruskal–Wallis test is therefore more appropriate for ordinal or skewed data, such as session counts and test frequencies observed in our dataset. The Kruskal-Wallis test produces an H-statistic and a p-value, where a p-value less than 0.05 suggests significant differences among group medians. Post-hoc analysis was performed using Dunn’s test for numerical variables that showed significant differences in the Kruskal-Wallis test. Dunn’s test facilitates pairwise comparisons between groups, with the results corrected for multiple testing using the Benjamini-Hochberg procedure to control the false discovery rate. For evaluations that involved tracking the same subjects across multiple timing events, we employed the Friedman test across various timing windows or before/after event comparisons. This non-parametric test is well-suited for data without normal distribution assumptions and repeated measures. Significant findings from the Friedman test led to pairwise comparisons using the Conover-Iman test, a non-parametric method appropriate for dependent samples. To control for multiple comparisons and maintain the overall error rate, p-values from the Conover-Iman test were adjusted using the Bonferroni correction.

General app usage patterns—including session duration, navigation frequency, and most visited pages—are reported in detail elsewhere [25]. In the present analysis, we focus specifically on paradata related to the HIV self-testing workflow, including test initiation, completion, and result interpretation behaviors.

### Ethical Considerations

The study was reviewed and approved by the Institutional Review Boards at Columbia University, Lurie Children’s Hospital in Chicago, and Vanderbilt University. The use of the mLab App was reviewed by the US Food & Drug Administration and assigned an Investigational Device Exemption (FDA IDE 18348).

## Results

Since assistance with, and analysis of, the OraQuick Self-Test was the primary purpose of the mLab App, we place particular emphasis on understanding the testing functionality of the application.

**Fig. 3:**
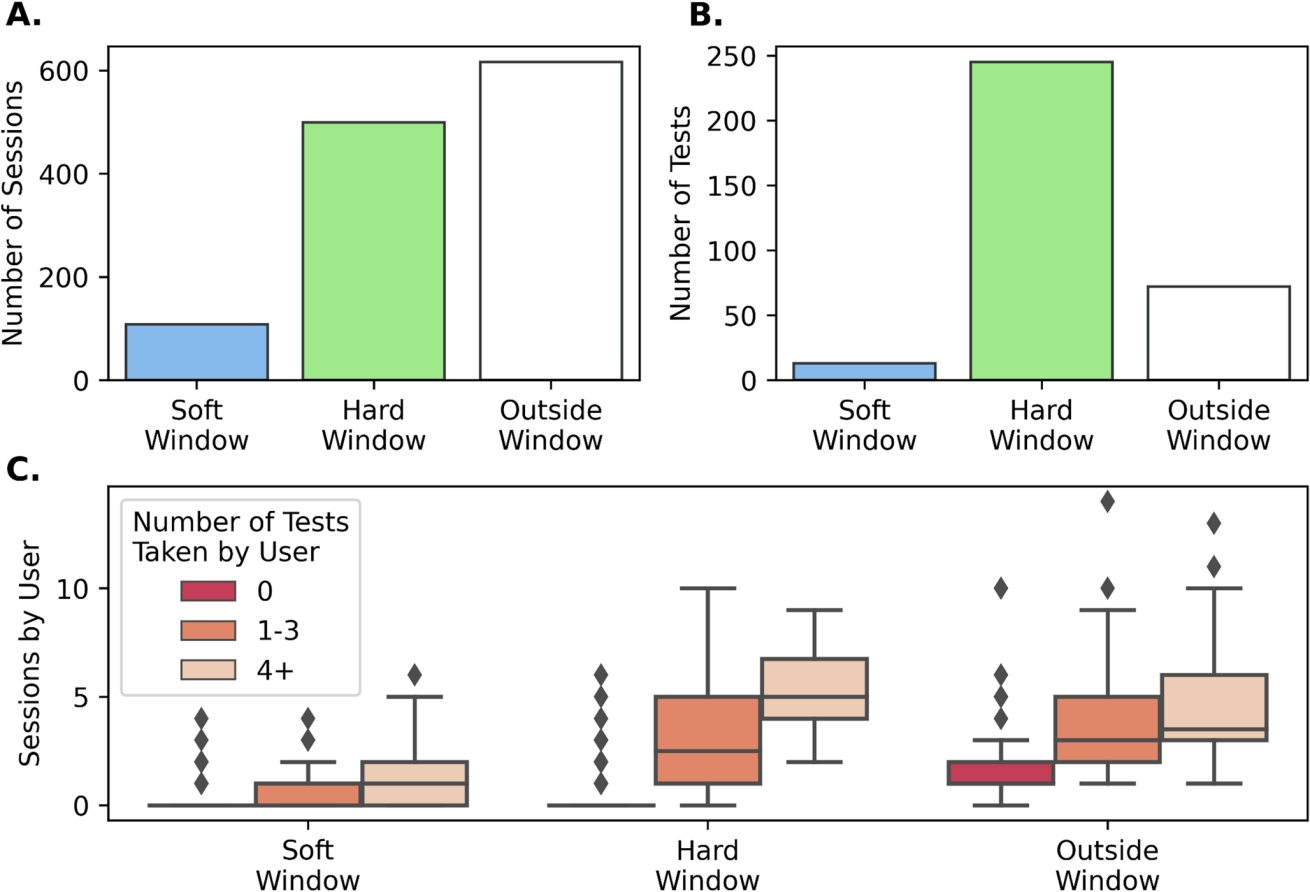
**A** The number of unique sessions inside and outside of the testing windows. **B** The number of tests performed inside and outside of the testing windows. **C** The number of sessions taken by users in each window, with users grouped by how many total tests a user took throughout their participation

While diagnostic accuracy of the mLab App has been described in detail elsewhere [24], here we primarily focus on how participants used the testing feature. Throughout the entire study, more sessions occurred outside (*n* = 616) of testing windows than within the soft window (*n* = 108) and hard window (*n* = 499) combined (Fig. 3A). However, 74.2% (*n* = 245) of the 330 tests taken with mLab were taken during the hard window; this is compared to 3.9% (*n* = 13) and 21.8% (*n* = 72) during the soft window and outside testing windows, respectively (Fig. 3B).

In our analysis, we categorized users based on the number of HIV self-tests they took, which allowed us to disaggregate total session counts into sessions per user as depicted in Fig. 3C. We observed that users who took more tests generally engaged more frequently with the application. Specifically, for the groups categorized as 0 tests, 1–3 tests, and 4 or more tests, session counts increased across the first quartile, median, and third quartile measurements.

We employed statistical tests to analyze session counts in different testing windows. Friedman tests indicated that, within each test-taking group, session activity differed across the soft, hard, and outside windows (0 tests: χ²(2) = 110.6, *p* < 0.001, Kendall’s W = 0.63; 1–3 tests: χ²(2) = 102.6, *p* < 0.001, W = 0.58; 4 + tests: χ²(2) = 41.8, *p* < 0.001, W = 0.61), indicating large within-person effects of the testing window. For users who took no tests, post-hoc comparisons showed that session counts were substantially higher outside the testing window than in either the hard or soft windows, with no meaningful difference between hard- and soft-window activity. Among participants who took 1–3 tests, soft-window activity was markedly lower than both hard-window and outside-window activity. For those taking four or more tests, session counts during the hard and outside windows were similar, but both were higher than in the soft window.

Using the Kruskal–Wallis test with post-hoc Dunn’s test, we also found significant differences in session distributions within the soft and hard windows across different test-taking groups. These effects were large for the hard window (H = 112.8, *p* < 0.001, η²_H = 0.54), medium–large for sessions outside the window (H = 71.6, *p* < 0.001, η²_H = 0.34), and small–to–medium for the soft window (H = 30.5, *p* < 0.001, η²_H = 0.14), indicating that users who ultimately completed more HIVSTs were also more active during those windows. In post-hoc analyses, session distributions during the soft window differed across all three test-taking groups, with users who completed 4 + tests showing the highest activity, followed by those who completed 1–3 tests, and then those who completed no tests. Similar graded patterns were observed in the hard and outside windows: users who ultimately completed more HIVSTs also generated more sessions, particularly compared with non-testers. Outside the testing window, users with 1–3 and 4 + tests showed comparable session counts, whereas both groups were more active than non-testers.

**Fig. 4:**
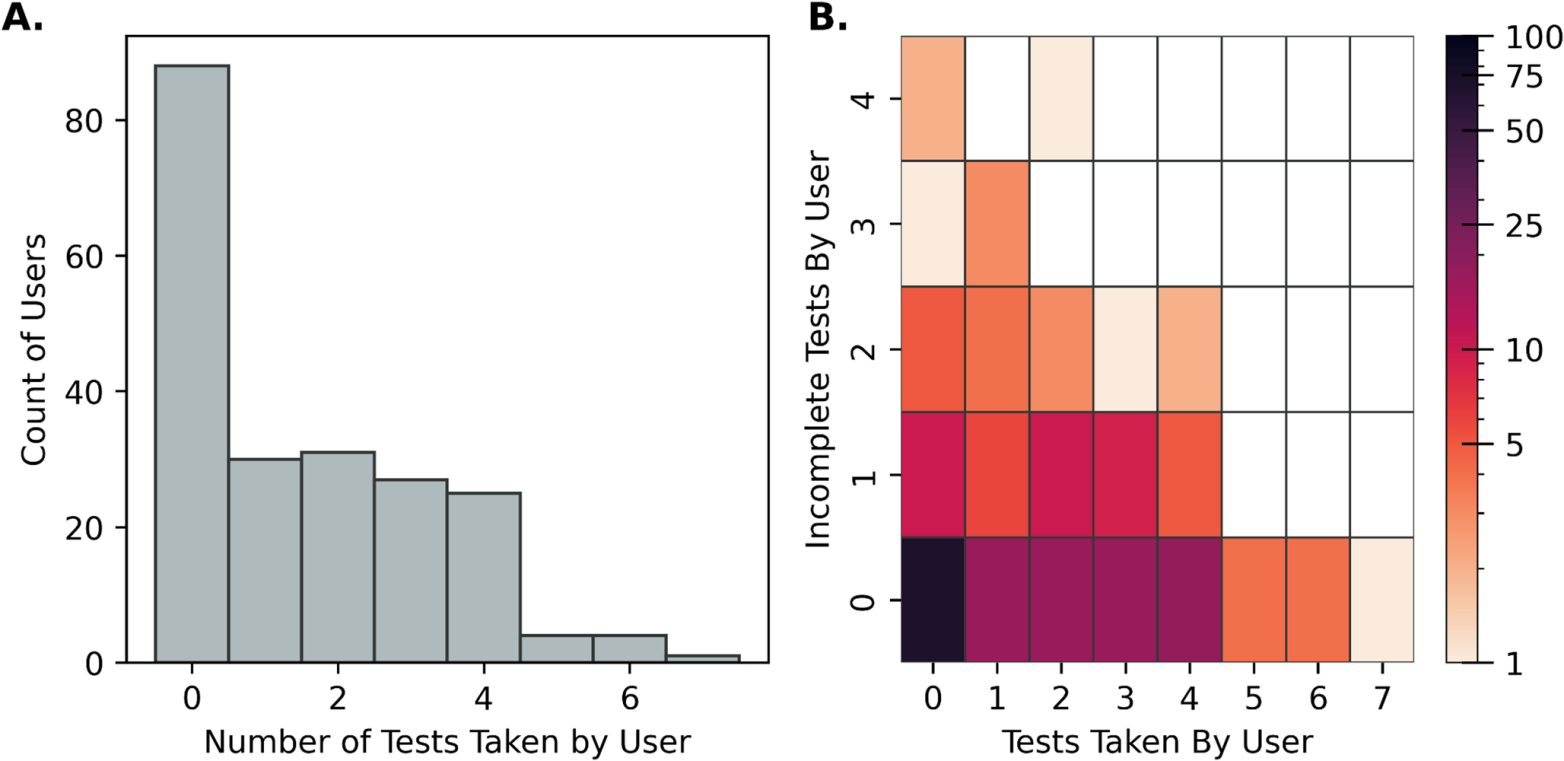
**A** Distribution of the number of tests taken by users. **B** The count of combinations of incomplete tests and completed tests taken by users

**Fig. 5:**
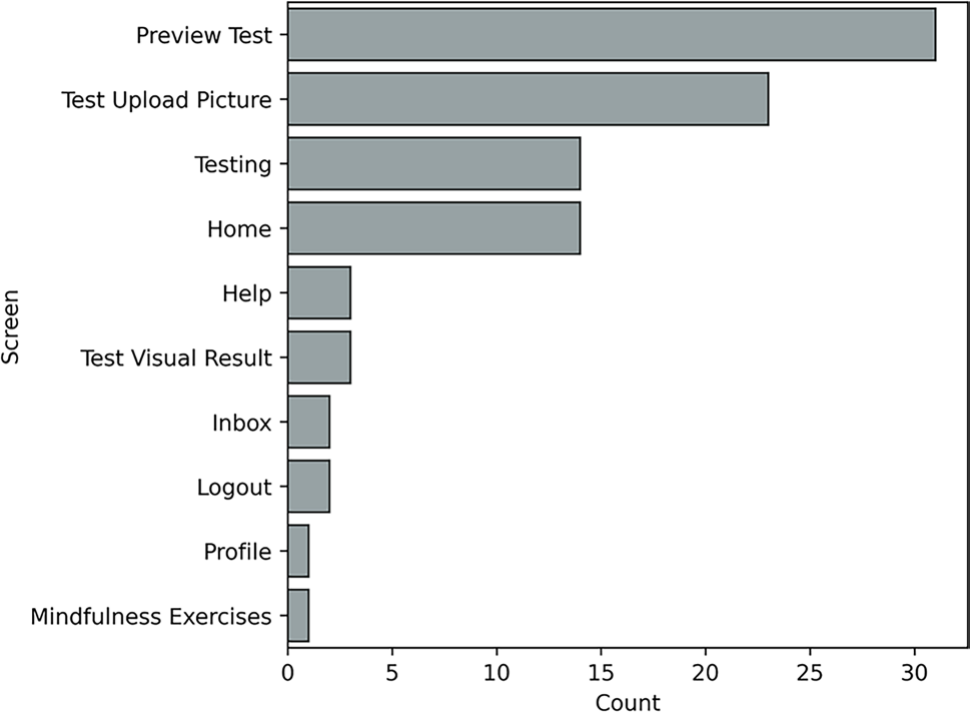
Terminal screens during incomplete testing sessions

Since completing HIV self-testing is the primary objective for users within the app, we look more closely at individual testing patterns. The median number of tests taken was 2.5 (*n* = 330); 88 users took no tests while 9 users took 5 or more tests (Fig. 4A). Using paradata, we are able to observe screen-level actions, allowing us to determine if a user initiated but did not complete a test (Fig. 4B). Of the 88 users who took no tests, 70 never initiated a test (0 incomplete, 0 tests taken), while 18 initiated between 1 and 4 tests without completing them. In contrast, 78 users were able to complete at least one test without having any incomplete tests. Ultimately, 50% (*n* = 105) users completed more tests than had incomplete tests; 37.6% (*n* = 79) users had an equal number of completed and incomplete tests (including the 70 users that never initiated a test); and 12.4% (*n* = 26) users had more incomplete tests than completed tests. Demonstrating the ability for paradata to bridge user-level, session-level, and screen-level data, we are able to analyze the terminal screens during incomplete testing sessions (Fig. 5). Three screens that occur after the HIVST timer is complete and the results are ready to be analyzed—Preview Test, Test Upload Picture, and Test Visual Result—correspond to the post-timer portion of the workflow shown in Fig. 1 (panels D–F), where users transition from waiting for results to photographing and confirming them. These screens accounted for 60.6% of incomplete testing sessions.

**Fig. 6:**
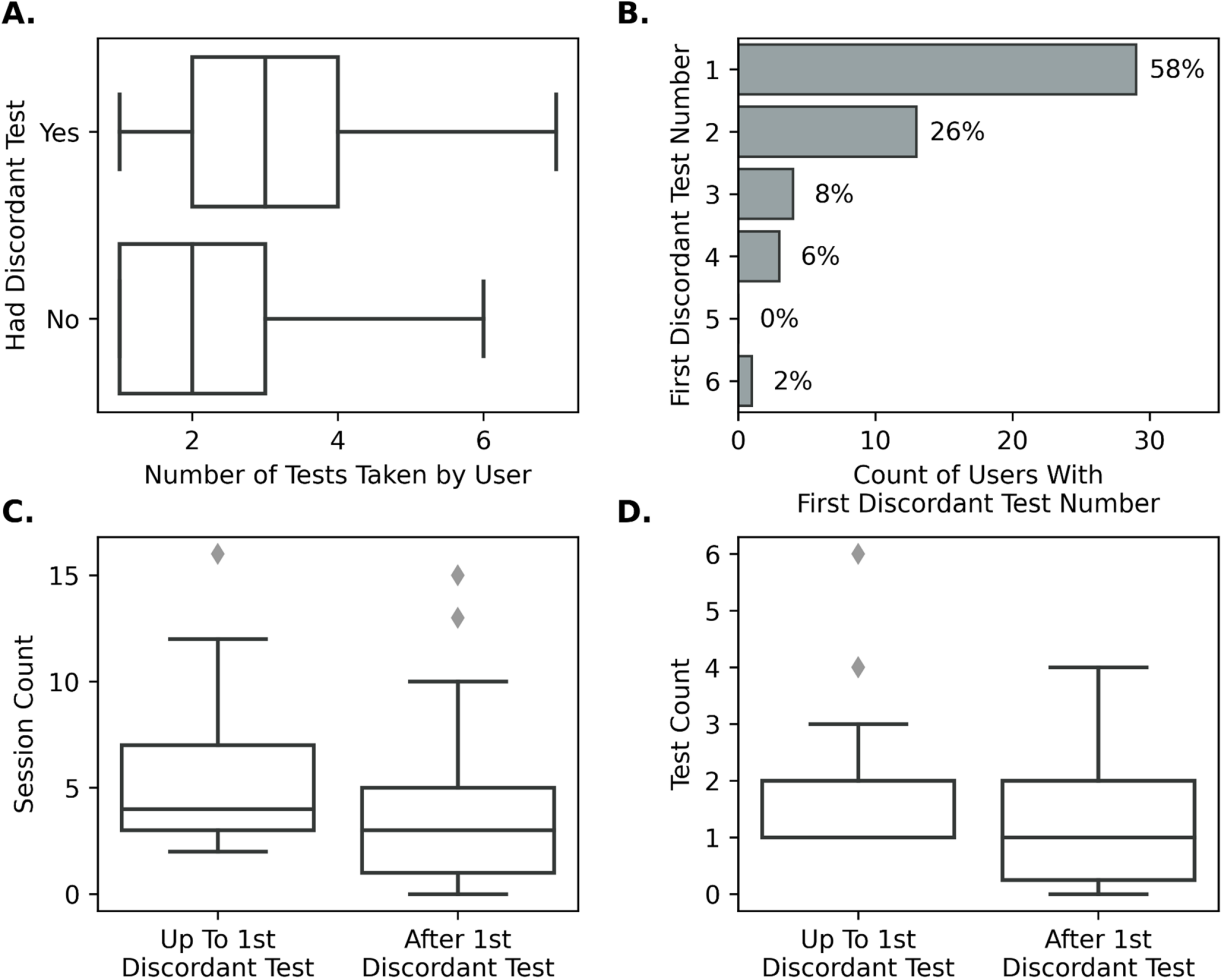
Tests and sessions of users that had a discordant test. **A** The distribution of tests taken by users that had and did not have discordant test results. **B** The count of when users had their first discordant test. **C** Of users that had discordant tests, the distribution of session counts before and after their first discordant test. **D** Of users that had discordant tests, the distribution of test counts before and after their first discordant test

With a companion app for HIVST, paradata can be particularly useful in understanding user behavior leading up to and after discordant results. Discordant results in our application were limited to false positives, where the experimental image processing algorithm indicated a positive test while the visual result reported by the participant and confirmed through visual inspection of the image by the study team, was a negative result. As noted in our other work [25], event-level paradata could be useful to determine whether users were aware of discordant results. In Fig. 6, we summarize when discordant results occurred for participants – and present high-level, aggregate data on whether that discouraged the users from taking future tests. From Fig. 6A, we can see that the more tests a user took, the more likely they were to experience at least one discordant result (Mann–Whitney U = 2273.0, Z = 2.61, *p* = 0.0076, *r* = 0.24, small-to-moderate). Most users (58%) that experienced a discordant test had it happen to them on their first test (Fig. 6B), and this percentage decreased as users’ test numbers increased. Of users that had a discordant test, the users had a higher median number of sessions leading up to their first discordant test than they did after their first discordant test (Fig. 6C; Wilcoxon W = 264.0, Z = − 2.70, *p* = 0.0069, *r* = 0.41, medium), indicating a short-term reduction in engagement after encountering a discordant digital interpretation. The median (1) and third quartile (2) number of tests prior to and after the first discordant test were identical and not significant (Fig. 6D; Wilcoxon W = 243.0, Z = − 1.18, *p* = 0.227, *r* = 0.20, small), suggesting that discordant results did not materially change longer-term HIVST behavior.

**Fig. 7:**
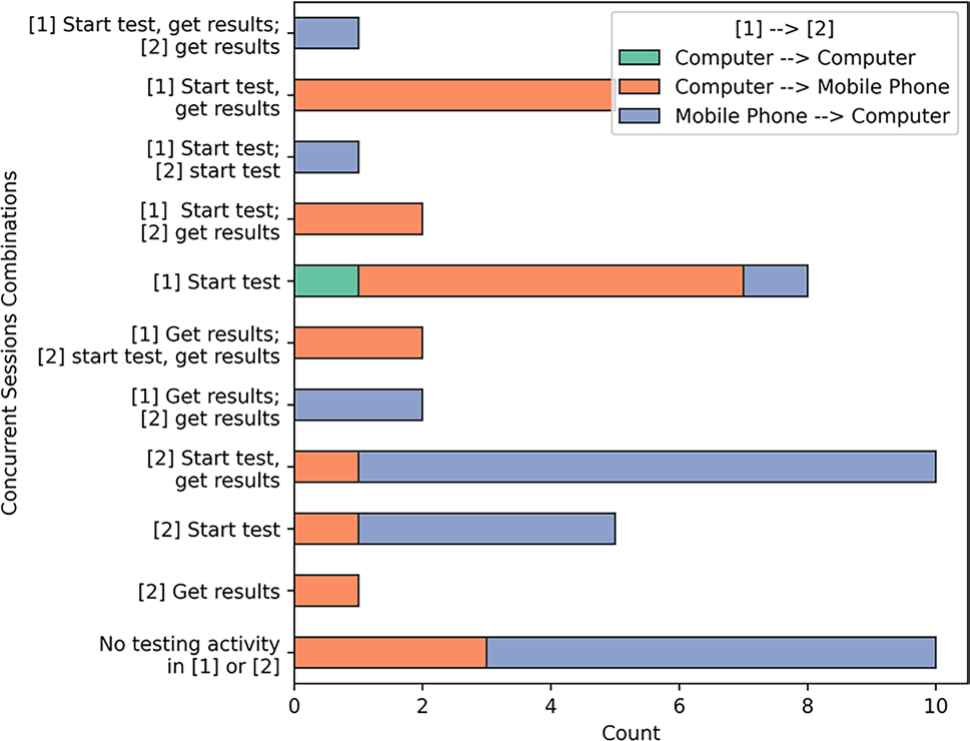
The count of sessions that started within 60 min of each other and began on one device [1] and ended on another [2], grouped by testing activity within each session

We previously demonstrated that some mLab App users accessed the application through multiple devices during their participation in the research study [25]. In this analysis, we observed that some users occasionally switched devices mid-session, transitioning from a phone to a computer or vice versa. Figure 7 visualizes device transitions between sequential sessions (defined as sessions initiated within 60 min of each other) in the mLab App, categorized by concurrent session combinations and user activities, such as starting a test, viewing results, or both. Each bar represents the count of specific combinations of activities across two sessions ([1] and [2]), with colors indicating the type of device transition (e.g., Computer to Mobile Phone or vice versa). The figure captures scenarios such as when no testing activity occurred in either session or when users completed activities like starting or concluding tests across devices. Certain combinations, such as “[2] Get results” or “[1] Start test,” reflect cases where only one session actively recorded testing-related actions while the other session remained idle or unrelated to testing. These scenarios emphasize that users often focus their testing activities on a single device or session, even when logged into multiple concurrent sessions. This behavior underscores the importance of tracking session activity to fully understand cross-device workflows and user engagement patterns. Notably, the figure also highlights a subset of users who switched devices during their interactions with the app, demonstrating how device transitions contribute to testing workflows. While this figure primarily visualizes transitions between two sessions, it is important to note that some users engaged in three or more concurrent sessions on different devices, where intermediate sessions acted as both succeeding and preceding connections. In a small number of sessions, we did observe users with multiple concurrent sessions on devices and tests that concluded in rapid succession.

## Discussion

HIV self-testing is often touted as a means to overcome concerns about stigma and privacy, and engage at-risk populations in the HIV continuum of care [5, 9, 10]. While this has mostly been true, the inherent privacy also brings a level of opacity in testing behavior and patterns. In this study, we leveraged paradata, detailed records of in-app usage, to understand behavior at the intersection of HIV self-testing and mobile health.

With the mLab App’s primary focus on assisting users with their HIV self-test, paradata enabled investigation into when and how often users signed in to complete a test. Although the majority of overall app sessions occurred outside of the recommended testing windows, most HIV self-tests were taken during the hard window, suggesting that reminders and notifications were effective in prompting timely test completion. This alignment between app-based prompts and testing behavior underscores the potential for digital nudges to reinforce adherence to recommended screening intervals—particularly in populations such as YMSM, where maintaining consistent HIV testing remains a challenge. Moreover, our analysis suggests that users who completed more tests also engaged more frequently with the app overall, indicating that higher engagement may reinforce adherence behaviors. Future work could explore adaptive reminder strategies that personalize test window notifications based on user engagement patterns, optimizing the balance between encouragement and alert fatigue.

A particularly notable finding was that three screens occurring after the HIV self-test timer—Preview Test, Upload Picture, and Visual Result—were responsible for 60.6% of incomplete testing sessions. Because these screens appear after the test’s result is visually apparent, they represent the point at which users transition from waiting to documenting and confirming their results. Several explanations may account for this pattern. Some users may have exited the app after visually inspecting their test, especially if they perceived a reactive result and preferred to process it privately before uploading the image or confirming the outcome. Others may have encountered technical or usability barriers, such as difficulty capturing a photo that met quality requirements. These potential friction points highlight a critical ‘last-mile’ challenge in the HIV self-testing workflow: users successfully perform the test but do not complete digital result submission. Addressing this gap could involve simplified post-timer steps, automated image capture with real-time feedback, or clearer messaging around privacy and linkage to confirmatory care. However, these explanations remain speculative, as our current paradata cannot distinguish the exact reasons for early exit. Further qualitative or event-level analysis would be needed to determine whether these behaviors were driven by emotional, technical, or contextual factors. Future work might also consider diagnostic devices that conceal the visual test result until after digital image capture, ensuring complete data submission while reducing user bias. Such designs, however, may introduce other trade-offs related to autonomy, user experience, and trust.

As with any diagnostic device, the mLab App can produce false positive and false negative results. Paradata allowed us to examine behavioral responses following discordant outcomes, specifically when the experimental image-processing algorithm produced a reactive (positive) interpretation that differed from the participant’s visual inspection. Although users tended to have fewer sessions after experiencing their first discordant result, this effect may partly reflect a ceiling effect among individuals who had already taken multiple tests. The similar number of tests before and after a discordant result suggests that overall testing frequency was not significantly affected, though short-term engagement may have been disrupted. These findings imply that momentary uncertainty introduced by discordant digital interpretations may dampen immediate engagement but does not necessarily discourage longer-term self-testing. In future app iterations, clearer on-screen messaging, visual aids, and linkage-to-care resources could help contextualize discordant results in real time—transforming potential confusion into an opportunity for education and reassurance.

This study has several limitations. First, the analysis was limited to paradata from participants in the mLab arm of a specific randomized clinical trial, which may restrict generalizability to other populations, app-based self-testing contexts, or digital health interventions. Second, while paradata capture provides granular behavioral data, it cannot directly infer user intent or emotional state during testing. These constraints inform opportunities for future studies incorporating mixed methods or real-time qualitative follow-up.

## Conclusions

Paradata provide a detailed view into the moment-to-moment behaviors that occur during HIV self-testing and can reveal critical points where users disengage from intended workflows. In this secondary analysis of a randomized clinical trial, screen-level paradata allowed us to characterize how participants progressed through the HIV self-test interpretation sequence, including when tests were initiated, where incomplete sessions occurred, and how users responded to discordant automated results. These findings demonstrate the value of paradata for identifying workflow bottlenecks and for informing the design of digital tools that support accurate HIV self-testing and linkage to care. Future work should incorporate mixed-methods approaches, real-time prompts, or enhanced event-level tracking to better understand why users exit at key stages of the workflow and how interface changes might mitigate those challenges. By refining digital support around result interpretation, HIV self-testing platforms may further improve usability, accuracy, and sustained engagement.

## Data Availability

The data that support the findings of this study are openly available at. 10.6084/m9.figshare.28211636.
